# Total pancreatectomy with islet autotransplantation reduces opioid use and improves nutritional support in children with debilitating pancreatitis

**DOI:** 10.1371/journal.pone.0289620

**Published:** 2023-08-04

**Authors:** Christie Heinzman, Lindsey Hornung, Tom K. Lin, Colleen M. O. Lowe, David S. Vitale, Maisam Abu-El-Haija, Jaimie D. Nathan

**Affiliations:** 1 Division of Pediatric General and Thoracic Surgery, Cincinnati Children’s Hospital Medical Center, Cincinnati, Ohio, United States of America; 2 Division of Biostatistics and Epidemiology, Cincinnati Children’s Hospital Medical Center, Cincinnati, Ohio, United States of America; 3 Division of Gastroenterology, Hepatology & Nutrition, Cincinnati Children’s Hospital Medical Center, Cincinnati, Ohio, United States of America; 4 Department of Pediatrics, University of Cincinnati College of Medicine, Cincinnati, Ohio, United States of America; Al-Azhar University, EGYPT

## Abstract

**Background:**

Chronic pancreatitis (CP) can result in opioid dependence and nutritional challenges in children. Total pancreatectomy with islet autotransplantation (TPIAT) is a viable surgical option in appropriately selected patients. We examined differences between children who met criteria for TPIAT versus those who did not and continued with non-operative management.

**Methods:**

Retrospective observational cohort study of patients evaluated for TPIAT between August 2014 and July 2020 was performed. Cohort-based analyses between TPIAT and non-TPIAT groups were performed.

**Results:**

Analyses included 121 patients, 69 of whom underwent TPIAT. Demographics, genetic risk factors, and anatomic variants did not differ between groups. TPIAT patients were more likely to have CP (88% vs 71%; *p* = 0.02), had higher median number of endoscopic retrograde cholangiopancreatography procedures (2.0 vs 1.0; *p* = 0.0001), and had higher likelihood of opioid use (61% vs 42%; *p* = 0.04) and nutritional supplementation (23% vs 4%; *p* = 0.004), compared to non-TPIAT. At 6 months post-TPIAT, patients had lower use of any analgesic pain medications (39% vs 73%; *p* = 0.0002) and lower use of opioids (9% vs 39%; *p* = 0.0006), compared to non-TPIAT patients at 6 months after evaluation. At 6 months post-TPIAT, rate of exclusively oral nutrition increased from 77% to 86%, and total parenteral nutrition use decreased from 13% to 0% (*p* = 0.02).

**Conclusions:**

In children referred for TPIAT evaluation, there is greater burden of disease in those selected for operation, compared to patients who do not undergo operation. TPIAT achieves lower analgesic pain medication use compared to continuation with non-TPIAT management and achieves freedom from nutritional supplementation.

**Level of evidence:** Retrospective comparative study, Level III.

## Introduction

Pancreatitis has been increasingly diagnosed in the pediatric population [1–3]. Studies report the incidence of childhood pancreatitis ranging from 3–13 cases per 100,000 persons per year, overlapping with the incidence in adult cohorts (5–60 cases per 100,000 persons per year), with cost of care exceeding $2.6 billion annually for inpatient care [4–6]. The increasing rate of diagnosis may be attributed to a greater awareness of pancreatitis in children with appropriate and timely diagnostic evaluation and clear definitions of disease [4]. The burden of acute recurrent pancreatitis (ARP) and chronic pancreatitis (CP) in children has substantial negative impact on quality of life with risks for long term consequences [7].

The etiology of pancreatitis in children is diverse and most commonly includes genetic risk factors, obstructive factors, and medications [8]. Treatment efforts are primarily focused on relieving pain and restoring quality of life, however, there are no definitive medical options to prevent recurrent pancreatitis episodes or to mitigate the progression to CP. Therapy is directed at managing symptoms and complications of disease, often with inclusion of therapeutic endoscopic procedures. When medical and endoscopic interventions have been exhausted and fail to relieve debilitation, surgical options may be considered [9].

Total pancreatectomy with islet autotransplantation (TPIAT) is a safe and effective surgical option for children with debilitating ARP or CP that has failed medical and endoscopic interventions [9–13]. First introduced in the 1970s in adults, TPIAT has now been successfully performed in hundreds of pediatric patients with the first case dating back to 1989 [14]. Selection for TPIAT involves a collaborative multidisciplinary decision-making model, and criteria slightly differ among institutions [12, 13, 15, 16]. Patients referred for TPIAT evaluation are assessed by a multidisciplinary team that includes pediatric gastroenterologists, surgeons, endocrinologists, pain management specialists, psychologists, radiologists, infectious disease specialists, nurses, dietitians, and social workers, among others. Some patients are deemed appropriate candidates for TPIAT as per the institution’s guidelines, while others are deferred. Overall, the degree of debilitation that occurs in patients with ARP or CP drives the decision-making process for candidacy. The recommendation for TPIAT is based on comprehensive review and assessment of the clinical course, radiologic findings, response to medications and interventions, and the current level of debilitation [10]. The baseline differences between the cohort of patients that meets criteria for TPIAT versus the cohort that does not requires further understanding, as does the clinical course following evaluation for TPIAT. The aims of this study are two-fold: (1) to assess the pre-evaluation differences between children who met criteria and proceeded to TPIAT (TPIAT group) versus children who did not meet criteria for TPIAT (non-TPIAT group), and (2) to assess the differences in opioid and nutritional outcomes in the TPIAT group versus non-TPIAT group at 6 months follow up.

## Methods

### Study design

A retrospective review of a prospectively collected institutional TPIAT database identified 121 patients who underwent TPIAT evaluation at a single, tertiary care center between August 2014 and July 2020. The study protocol was reviewed and approved by the Institutional Review Board (IRB) #2020–0854. The IRB deemed the study exempt and waived the need for consent. All patients who were evaluated for TPIAT during the study period were included in this study, and analyses were performed comparing the cohort that underwent TPIAT (TPIAT group) versus the cohort that did not undergo TPIAT (non-TPIAT group). Referrals for TPIAT evaluation were made by providers within and external to the institution. The center’s criteria utilized for consideration for TPIAT were published previously [12]. In brief, patients must have pain consistent with pancreatic origin or debilitation from pancreatitis for at least 6 months, as demonstrated by either chronic opioid dependence or impaired quality of life (frequent school absences and/or hospitalizations). Data obtained from the database included demographics, etiology and classification of pancreatitis, age of onset, surgical and endoscopic history, genetic risk factors, as well as baseline and follow-up data on endocrine and exocrine function, nutritional status and opioid consumption. Not all patients had the same number of genes tested, as genetic testing was performed at provider discretion with most patients being screened for at least the four most common genes (PRSS1, CFTR, SPINK1, CTRC). Data were collected at baseline during the multidisciplinary evaluation and again at 6 months following TPIAT operation in the TPIAT group and at 6 months after evaluation in the non-TPIAT group. Not all patients in the non-TPIAT group returned to our institution for follow-up at 6 months and therefore were excluded from the 6-month follow-up analyses.

### Disease classification

Disease was classified using standardized definitions from the INternational Study Group of Pediatric Pancreatitis: In search for a cuRE (INSPPIRE) consortium. ARP was diagnosed in patients with more than one episode of acute pancreatitis, regardless of severity, with complete normalization of amylase and lipase between episodes or complete resolution of pain between episodes [17]. CP was diagnosed in the presence of at least one of the following: (1) typical abdominal pain and characteristic imaging findings, (2) exocrine pancreatic insufficiency and characteristic imaging findings, (3) endocrine insufficiency and characteristic imaging findings, or (4) a surgical or pancreatic biopsy demonstrating histopathology features compatible with chronic pancreatitis [17].

### Surgical procedure

TPIAT operation was performed as previously published [18]. In brief, TPIAT consists of total pancreatectomy, near-total duodenectomy, splenectomy, cholecystectomy, appendectomy and a pylorus-preserving Roux-en-Y gastrointestinal reconstruction with Roux-en-Y choledochojejunostomy or hepaticojejunostomy. Following pancreatectomy, islets are enzymatically and mechanically isolated from the pancreas for autotransplantation into the liver via the portal vein during the operation. Prior to the islet autotransplantation, a sample of the islet isolation product was taken to calculate islet mass (islet equivalents/kg body weight). Islets were autotransplanted regardless of the number of islet equivalents/kg body weight, as there was no minimum threshold required for the release of the isolation product for infusion. The process of pancreatic islet isolation has been previously described by this group [19].

### Statistical analysis

Data were analyzed using SAS, version 9.4 (SAS Institute, Cary, NC). Continuous data were summarized as median values with interquartile ranges (IQR: 25^th^-75^th^ percentiles), and categorical data were summarized as frequency counts and percentages. Categorical data were analyzed using Chi-square or Fisher’s exact tests, as appropriate, and continuous data compared using Mann-Whitney-Wilcoxon tests. McNemar’s tests were used to analyze categorical paired comparisons. A *p* value of <0.05 was considered statistically significant. A subset of these patients was published in other studies with different study design and divergent goals and aims [10, 20–27]. A subset of these patients was enrolled in the Consortium for the Study of Chronic Pancreatitis, Diabetes and Pancreatic Cancer (CPDPC) and in the Prospective Observational Study of TPIAT (POST) multicenter study, also under different goals and objectives.

## Results

### Demographics

Between August 2014 and July 2020, 121 patients were evaluated for TPIAT and are included in this study. Sixty-eight females (56%) and 53 males (44%) underwent evaluation for TPIAT at a median age of 12.1 years (IQR: 9.2–14.6). Demographics including age, sex, height, weight and BMI percentile were not significantly different between the TPIAT (n = 69) and non-TPIAT (n = 52) groups (Table 1). In the entire cohort of patients evaluated for TPIAT, the median ages at 1^st^ and 2^nd^ acute pancreatitis (AP) attacks were 8.0 and 9.4 respectively, with progression to CP at a median of 10.3 years of age (Table 2). There was a significantly higher proportion of CP in the TPIAT group (88%), compared to the non-TPIAT group (71%; *p* = 0.02). The median number of AP attacks was higher in the TPIAT group (8.5, IQR: 6.0–12.0), compared to the non-TPIAT group (6.5, IQR: 3.0–10.0), but the difference did not reach statistical significance (*p* = 0.07). At the time of TPIAT evaluation, exocrine insufficiency (TPIAT group 48% versus non-TPIAT group 52%, *p* = 0.64) and endocrine function measured by baseline hemoglobin A1c (TPIAT group median value 5.2 [IQR: 5.0–5.4] versus non-TPIAT group median value 5.2 [IQR: 4.9–5.4]; *p =* 0.82) were not statistically different between the groups.

**Table 1:** Demographics, genetic risk factors, and anatomic variants.

	All n = 121	Non-TPIAT group n = 52	TPIAT group n = 69	*p* value
**Age at evaluation (years)**	12.1 (9.2–14.6)	11.6 (9.7–13.4)	12.9 (8.6–14.7)	0.54
**Age at TPIAT (years)**	13.1 (8.9–15.1)	-	13.1 (8.9–15.1)	-
**Time from evaluation to TPIAT (months)**	2.8 (1.9–4.4)	-	2.8 (1.9–4.5)	-
**Time from evaluation to follow-up (years)**	2.3 (1.3–3.7)	2.4 (1.3–3.8)	2.3 (1.2–3.4)	0.42
**Sex (female)**	68 (56%)	26 (50%)	42 (61%)	0.23
**BMI percentile**	77.4 (48.2–94.1)	72.4 (49.4–96.7)	79.3 (47.5–93.2)	0.88
**Weight percentile**	71.7 (38.4–94.3)	67.0 (35.8–95.7)	72.7 (42.5–91.9)	0.71
**Height percentile**	49.7 (26.0–74.5)	50.9 (25.7–66.6)	48.1 (26.0–76.8)	0.58
**Sweat chloride** **Negative** **Positive (30–60)** **Positive (>60)**	55/66 (83%) 7/66 (11%) 4/66 (6%)	23/26 (88%) 3/26 (12%) 0/26 (0%)	32/40 (80%) 4/40 (10%) 4/40 (10%)	0.28
**Positive genetic testing**	91 (75%)	41 (79%)	50 (72%)	0.42
** PRSS1**	39/118 (33%)	20/51 (39%)	19/67 (28%)	0.21
** SPINK1**	32/108 (30%)	13/47 (28%)	19/61 (31%)	0.69
** CFTR**	36/107 (34%)	15/47 (32%)	21/60 (35%)	0.74
** CTRC**	9/93 (10%)	2/40 (5%)	7/53 (13%)	0.29
** CASR**	0/64 (0%)	0/28 (0%)	0/36 (0%)	1.00
** CEL**	0/59 (0%)	0/25 (0%)	0/34 (0%)	1.00
** CLDN2**	0/56 (0%)	0/25 (0%)	0/31 (0%)	1.00
** CPA1**	1/57 (2%)	0/25 (0%)	1/32 (3%)	1.00
** SBDS**	0/56 (0%)	0/25 (0%)	0/31 (0%)	1.00
** UBR1**	1/56 (2%)	1/25 (4%)	0/31 (0%)	0.45
** CFTR + another gene**	22/107 (21%)	8/47 (17%)	14/60 (23%)	0.42
** CFTR + SPINK1**	14/105 (13%)	5/46 (11%)	9/59 (15%)	0.57
** CFTR + PRSS1**	5/105 (5%)	3/46 (7%)	2/59 (3%)	0.65
** SPINK1 + PRSS1**	2/105 (2%)	1/46 (2%)	1/59 (2%)	1.00
**More than 1 gene positive**	24/109 (22%)	10/48 (21%)	14/61 (23%)	0.79
**Anatomic variant present**	33 (27%)	10 (19%)	23 (33%)	0.08
**Pancreas Divisum** **Complete** **Incomplete**	33 (27%) 16/33 (48%) 17/33 (52%)	10 (19%) 6/10 (60%) 4/10 (40%)	23 (33%) 10/23 (43%) 13/23 (57%)	0.08 0.46
**Annular pancreas**	1 (1%)	0 (0%)	1 (1%)	1.00
**Divisum + genetic mutation** **Divisum + PRSS1** **Divisum + SPINK1** **Divisum + CFTR**	22 (18%) 11/118 (9%) 8/108 (7%) 4/107 (4%)	7 (13%) 4/51 (8%) 3/47 (6%) 0/47 (0%)	15 (22%) 7/67 (10%) 5/61 (8%) 4/60 (7%)	0.24 0.76 1.00 0.13
**Annular pancreas + genetic mutation**	1 (1%)	0 (0%)	1 (1%)	1.00

Data presented as median (25^th^-75^th^ percentile) or n (%).

BMI: body mass index; TPIAT: total pancreatectomy with islet autotransplantation

**Table 2:** Pancreatitis course and interventions.

	All n = 121	Non-TPIAT group n = 52	TPIAT group n = 69	*p* value
**Age at 1**^**st**^ **AP attack (years)**	8.0 (4.5–11.2)	7.3 (4.2–10.3)	9.3 (4.6–11.8)	0.18
**Age at 2**^**nd**^ **AP attack (years)**	9.4 (5.9–12.3) *n = 97*	8.4 (5.9–11.4) *n = 34*	10.4 (5.5–12.7) *n = 63*	0.48
**Number of AP attacks**	8.0 (5.0–12.0) *n = 98*	6.5 (3.0–10.0) *n = 36*	8.5 (6.0–12.0) *n = 62*	0.07
**Pancreatitis status at TPIAT evaluation** **ARP** **CP**	23 (19%) 98 (81%)	15 (29%) 37 (71%)	8 (12%) 61 (88%)	**0.02**
**Age at CP diagnosis (years)**	10.3 (6.1–13.7) *n = 98*	9.6 (6.4–12.7) *n = 36*	10.9 (5.6–14.1) *n = 62*	0.91
**Duration CP to TPIAT evaluation (months)**	8.2 (2.3–27.9) *n = 98*	5.7 (1.1–26.6) *n = 36*	9.3 (3.1–28.1) *n = 62*	0.26
**# ERCPs prior to TPIAT evaluation**	2.0 (1.0–3.0)	1.0 (1.0–2.0)	2.0 (1.0–4.0)	**0.0001**
**Prior cholecystectomy**	15 (12.4%)	5 (9.6%)	10 (14.5%)	0.58
**Prior pancreas operation**	8 (6.6%)	3 (5.8%)	5 (7.2%)	1.00

Data presented as median (25^th^-75^th^ percentile) or n (%).

AP: acute pancreatitis; ARP: acute recurrent pancreatitis; CP: chronic pancreatitis; ERCP: endoscopic retrograde cholangiopancreatography; TPIAT: total pancreatectomy with islet autotransplantation

### Genetic risk factors

Genetic risk factors were found in 75% (n = 91) of the entire cohort with no difference between the TPIAT group (72%, n = 50) versus the non-TPIAT group (79%, n = 41; *p* = 0.42) (Table 1). The *CFTR* gene was the most common to have variants detected in the TPIAT group (35%, 21/60), while in the non-TPIAT group, *PRSS1* was the most common genetic risk factor found (39%, 20/51). Overall, 24 patients (22%) had more than one genetic risk factor identified, but this did not differ between the two groups (*p* = 0.79; Table 1).

### Anatomic variants

Patients were screened for anatomic variants including pancreas divisum and annular pancreas, with pancreas divisum being further classified as complete or incomplete. Magnetic resonance cholangiopancreatography (MRCP) and endoscopic retrograde cholangiopancreatography (ERCP) were used to evaluate for the presence and type of pancreas divisum. Pancreas divisum was found in 27% (n *=* 33) of the entire cohort (TPIAT group 33% [n = 23] versus non-TPIAT group 19% [n = 10]; *p* = 0.08) (Table 1). There was no statistical difference between the TPIAT group and the non-TPIAT group in terms of the proportion of pancreas divisum that was complete versus incomplete (*p* = 0.46). The presence of pancreas divisum plus at least one genetic risk factor was found in 18% (n = 22) of the overall cohort (TPIAT group 22% [n = 15] versus non-TPIAT group 13% [n = 7]; *p* = 0.24). In the entire cohort, the most common genetic mutation found associated with pancreas divisum was *PRSS1* in 9% of patients (11/118). Annular pancreas in combination with SPINK1 mutation was found in one patient, who proceeded to TPIAT.

### Procedural history

The median number of ERCPs at the time of evaluation was significantly higher in TPIAT group (2.0, IQR: 1.0–4.0) compared to the non-TPIAT group (1.0, IQR: 1.0–2.0; *p* = 0.0001) (Table 2). In the overall cohort of patients undergoing TPIAT evaluation, 12.4% underwent prior cholecystectomy and 6.6% had undergone a prior pancreas operation, including Puestow procedure, Whipple procedure, distal pancreatectomy, and transduodenal sphincteroplasty of the minor papilla, but the differences between the TPIAT group and the non-TPIAT group did not reach statistical significance.

### Opioid use and nutritional support

There were significant differences between the non-TPIAT group and the TPIAT group in terms of opioid use within the year prior to evaluation and the need for supplemental nutrition at evaluation (Table 3). In the TPIAT group, a higher proportion of patients required both opioid and non-opioid analgesic medications (48% vs 21%), while in the non-TPIAT group, a higher proportion of patients required non-opioid analgesic medications only (37% vs 29%) or did not require any analgesic pain medications (21% vs 10%) (*p* = 0.02). Any opioid use, including opioids alone or in combination with a non-opioid medication, was significantly more common in the TPIAT group (61%; 42/69), compared to the non-TPIAT group (42%, 22/52; *p* = 0.04). Regarding baseline nutrition, enteral nutrition (EN) or total parenteral nutrition (TPN) supplementation was required in 23% (16/69) of patients in the TPIAT group at time of evaluation, compared to only 4% (2/52) in the non-TPIAT group (*p* = 0.004). Micronutrient levels, including fat soluble vitamins, iron stores, and vitamin B12 were also analyzed at evaluation, but there were no statistical differences between the two groups.

**Table 3:** Pain medication use and nutritional support at time of TPIAT evaluation.

	All n = 121	Non-TPIAT group n = 52	TPIAT group n = 69	*p* value
**Analgesic pain medication use in last year** **None** **Opioid use only** **Non-opioid use only** **Both opioid and non-opioid use**	18 (15%) 20 (17%) 39 (32%) 44 (36%)	11 (21%) 11 (21%) 19 (37%) 11 (21%)	7 (10%) 9 (13%) 20 (29%) 33 (48%)	**0.02**
**Any opioid use in last year**	64 (53%)	22 (42%)	42 (61%)	**0.04**
**Frequency of opioid use in last year** **Daily** **<3 times/week**	19/64 (30%) 45/64 (70%)	5/22 (23%) 17/22 (77%)	14/42 (33%) 28/42 (67%)	0.57
**Nutrition method** **PO only** **TPN (any)** **EN (only or PO + EN)**	103 (85%) 9 (7%) 12 (10%)	50 (96%) 0 (0%) 2 (4%)	53 (77%) 9 (13%) 7 (10%)	**0.004**

Data presented as n (%).

EN: enteral nutrition; PO: per os; TPN: total parenteral nutrition

Six months following TPIAT operation, there was significantly lower use of any analgesic pain medications in the TPIAT group (61% requiring no analgesics), compared to analgesic use 6 months following evaluation in the non-TPIAT group (27% requiring no analgesics; *p* = 0.0002; Table 4). Only 9% of TPIAT patients were requiring opioids at 6 months after operation, compared to 39% of patients in the non-TPIAT group requiring opioids at 6 months following evaluation for TPIAT (*p* = 0.0006). Following TPIAT operation, there was a significant reduction in the use of analgesic pain medications, as well as improvement in the reliance on supplemental nutrition, in the TPIAT group 6 months post-operatively (Table 5). For the TPIAT group, there was a significant decrease in the proportion of patients with any opioid use from 61% to 9% (*p*<0.0001) at 6 months. The percent of patients taking exclusively oral nutrition increased from 77% to 86%, and TPN use was weaned from 13% to 0% (*p* = 0.02), with the increase in enteral supplementation from 10% to 14% encompassing children who were TPN-dependent prior to TPIAT. For the non-TPIAT group, at 6 months after evaluation, there was no significant change in analgesic pain medication use and no change in nutritional support, with a single patient requiring enteral supplementation.

**Table 4:** Pain medication use at 6 months post-evaluation or 6 months post-TPIAT.

	Non-TPIAT group n = 33	TPIAT group n = 69	*p* value
**Analgesic pain medication use at 6 months post** **None** **Opioid use only** **Non-opioid use** **Both opioid and non-opioid use**	9 (27%) 1 (3%) 11 (33%) 12 (36%)	42 (61%) 2 (3%) 21 (30%) 4 (6%)	**0.0002**
**Any opioid use at 6 months post**	13 (39%)	6 (9%)	**0.0006**

Data presented as n (%).

**Table 5:** Pain medication and nutritional support at evaluation compared to 6 months post-evaluation or 6 months post-TPIAT.

	Non-TPIAT group			TPIAT group		
	**Eval** **n = 33**	**6 months post** **n = 33**	^#^ ***p* value**	**Eval** **n = 69**	**6 months post** **n = 69**	^#^ ***p* value**
**Analgesic pain medication use** **None** **Opioid use only** **Non-opioid use only** **Both opioid and non-opioid use**	5 (15%) 8 (24%) 10 (30%) 10 (30%)	9 (27%) 1 (3%) 11 (33%) 12 (36%)	0.26	7 (10%) 9 (13%) 20 (29%) 33 (48%)	42 (61%) 2 (3%) 21 (30%) 4 (6%)	**<0.0001**
**Any opioid use**	18 (55%)	13 (39%)	0.10	42 (61%)	6 (9%)	**<0.0001**
**Nutrition method** **PO only** **TPN any** **EN (only or PO + EN)**	32 (97%) 0 (0%) 1 (3%)	32 (97%) 0 (0%) 1 (3%)	1.00	53 (77%) 9 (13%) 7 (10%)	59 (86%) 0 (0%) 10 (14%)	**0.02**

Data presented as n (%).

EN: enteral nutrition; PO: per os; TPN: total parenteral nutrition

^#^Represents comparison between Eval and 6 months post

## Discussion

Children with ARP and CP are at risk for chronic pain, opioid dependence and nutritional challenges. Patients who remain debilitated despite medical and endoscopic interventions can be evaluated for TPIAT, however, not all will be suitable candidates. This study is the first to analyze a large pediatric cohort of patients who underwent evaluation for TPIAT, reporting on the differences between children who met criteria and proceeded to TPIAT versus children who did not meet criteria for TPIAT after multidisciplinary evaluation. We have shown that children undergoing TPIAT are more likely to have a diagnosis of CP and to have undergone a greater number of ERCPs, as well as to have a greater opioid burden and a greater need for nutritional supplementation. In addition, we have shown that children undergoing TPIAT achieve a lower opioid burden compared to children who continue with non-TPIAT management approaches and that nutritional supplementation with TPN is relieved at 6 months postoperatively.

Our findings are novel in that they demonstrate the specific preoperative disease burden of children selected for TPIAT. A study from the INSPPIRE consortium has previously reported a 28% rate of opioid use in a cohort of children with ARP or CP, with 77% of children reporting pain in the year prior to enrollment, although it must be recognized that INSPPIRE enrolls all patients with CP or ARP [28]. Opioid use in 62% of the TPIAT group in our study is not surprising, as our cohort represents those children who are the most severely affected by their disease and have failed medical and endoscopic interventions. Notably, opioid dependence is not a required criterion to undergo TPIAT. Although a previous study of a cohort of children who underwent TPIAT reported that all patients were on opioids before operation [29], it is important to recognize that an additional critical component of disease burden in children is represented by inability to maintain nutrition independently. Herein, we have reported that 23% of our cohort required nutritional supplementation to meet their needs and support appropriate growth with either TPN and/or enteral tube feedings prior to operation. A number of institutions have previously published criteria for consideration of TPIAT [12, 13, 15, 16], and while there are some nuances between institutions regarding the definition of “debilitation,” it is important to note that children may face significant nutritional debilitation due to their disease. Additionally, we report a rapid progression (within approximately 2 years) in our cohort from first AP attack to the diagnosis of CP. While, again, our cohort of patients represents a select population of severely affected children, it highlights the importance of early referral of children with ARP or CP to centers with multidisciplinary pancreas expertise to potentially allow any medical and/or endoscopic interventions that may slow disease progression.

In appropriately selected patients, TPIAT provides pain relief in 90–95% of pediatric patients, and studies report opioid independence following pediatric TPIAT as high as 80% at one year postoperatively [29]. Bellin et al. evaluated outcomes in 17 patients aged 3–8 years undergoing TPIAT for CP and reported that all patients had improvement in pain and were completely weaned off opioids at one year postoperatively [30]. In a larger pediatric cohort, significant improvement in health-related quality of life scores was reported at one-year post-TPIAT [29]. We have also previously published on a subset of this TPIAT cohort, reporting improvement in opioid use and decreased need for supplemental nutrition within 90 days after TPIAT [10]. The results of the current study confirm previous publications regarding improvement in nutrition and opioid use following TPIAT operation. Our current results also demonstrate a continued trend of decreased opioid use and supplemental nutrition extending from our previously reported 90-day outcome data to 6 months following operation. Importantly, we demonstrate that the opioid burden at 6 months following TPIAT is significantly lower than the opioid burden in children who continue with non-surgical therapy after evaluation for TPIAT. Nevertheless, longitudinal outcome studies are needed to better understand the long-term outcomes following pediatric TPIAT.

While we report novel outcomes in this study, it does have limitations. It is a single center cross-sectional review of prospectively collected data of pediatric patients undergoing TPIAT evaluation. Our sample size for this study was small, which may have affected our ability to detect statistically significant findings across other variables. However, even with a small sample size, we were able to show significance in the difference between the groups and significant improvements following TPIAT in relation to opioid dependence and reliance on supplemental nutrition. In addition, the analgesic use at 6 months following evaluation in the non-TPIAT group was not differentiated between ongoing outpatient use versus inpatient use during acute pancreatitis exacerbations. Lastly, not all patients who were evaluated and not offered surgical intervention returned to our institution for follow up which affected our follow-up data in the non-TPIAT group.

In conclusion, pancreatitis in children is increasing in frequency causing debilitation that cannot be adequately managed by medical and endoscopic modalities in a subset of patients with ARP or CP. For these patients, TPIAT can be offered as an option leading to independence from opioids and minimization of supplemental nutrition. Further understanding of the criteria for patient selection will ensure optimization of post-TPIAT outcomes. Future larger multicenter studies are still needed to better understand the long-term outcomes after TPIAT.

## Abbreviations

AP: acute pancreatitis
ARP: acute recurrent pancreatitis
BMI: body mass index
CP: chronic pancreatitis
CPDPC: Consortium for the Study of Chronic Pancreatitis, Diabetes and Pancreatic Cancer
EN: enteral nutrition
ERCP: endoscopic retrograde cholangiopancreatography
INSPPIRE: INternational Study Group of Pediatric Pancreatitis: In search for a cuRE
IQR: interquartile range
MRCP: magnetic resonance cholangiopancreatography
POST: Prospective Observational Study of TPIAT
TPIAT: total pancreatectomy with islet autotransplantation
TPN: total parenteral nutrition
